# Striatal Input- and Rate-Dependent Effects of Muscarinic Receptors on Pallidal Firing

**DOI:** 10.1100/2012/547638

**Published:** 2012-05-01

**Authors:** Enrique Querejeta, Alberto Alatorre, Alain Ríos, Rafael Barrientos, Aldo Oviedo-Chávez, Rosa Amalia Bobadilla-Lugo, Alfonso Delgado

**Affiliations:** ^1^Sección de Estudios de Posgrado e Investigación, Escuela Superior de Medicina, Instituto Politécnico Nacional, Plan de San Luis y Díaz Mirón, 11340 México, DF, Mexico; ^2^Departamento de Fisiología, Facultad de Medicina Universidad Autónoma de Chihuahua, Circuito Universitario Campus II, 31125 Chihuahua, Chih, Mexico

## Abstract

The globus pallidus (GP) plays a key role in the overall basal ganglia (BG) activity. Despite evidence of cholinergic inputs to GP, their role in the spiking activity of GP neurons has not received attention. We examine the effect of local activation and blockade of muscarinic receptors (MRs) in the spontaneous firing of GP neurons both in normal and ipsilateral striatum-lesioned rats. We found that activation of MRs produces heterogeneous responses in both normal and ipsilateral striatum-lesioned rats: in normal rats the response evoked by MRs depends on the predrug basal firing rate; the inhibition evoked by MRs is higher in normal rats than in striatum-lesioned rats; the number of neurons that undergo inhibition is lower in striatum-lesioned rats than in normal rats. Our data suggest that modulation of MRs in the GP depends on the firing rate before their activation and on the integrity of the striato-pallidal pathway.

## 1. Introduction

The use of antimuscarinic drugs in the management of Parkinson's disease either as monotherapy or as an adjunct to other drugs is still in wide practice [1, 2]. The initial concepts of BG function have pointed out to the striatum as the target of antimuscarinic drugs to relief Parkinsonian motor symptoms [3, 4]. However, the presence of MRs modifies the activity of other BG nuclei [5–7].

The GP plays a key role in the global activity of basal ganglia [8]. Variations in the basal spontaneous firing rate of pallidal neurons are related to movement disorders both in humans and in animal models of Parkinson's disease [9, 10]. The effect of a wide variety of neurotransmitters on the electrical activity of GP neurons has been studied [11–16], yet, despite the presence of cholinergic fibers in the GP coming from the pedunculopontine nucleus (PPN) [17–20], there are no studies on the effect of acetylcholine on the spiking activity of GP neurons.

Previous works have shown the presence of MRs in the GP [21, 22] but studies of *in situ* hybridization and single-cell reverse transcription-polymerase chain reaction techniques have not detected mRNA for MRs in GP neurons. In addition, mRNA for M1 and M4 receptors has been found in medium spiny striatal neurons [23–25] projecting to the GP. In this context, Kayadjanian et al. [26] demonstrated that activation of MRs increases GABA release in GP rat brain slices and that this effect could be blocked by a muscarinic antagonist. These data pointed us to examine the regulation of GABA release in the GP by presynaptic striato-pallidal MRs. Therefore, here we analyze the effect of the local activation and blockade of MRs on the spontaneous spiking rate of GP neurons in both normal and ipsilateral striatum-lesioned rats.

## 2. Material and Methods

Experiments were performed on male Wistar rats weighing 180–220 g. Rats were maintained and handled according to the guidelines of the ESM-IPN Animal Care and Use Committee and followed the Guide for the Care and Use of Laboratory Animals published by the U.S. National Institutes of Health.

### 2.1. Surgery and Drug Application

Rats were anaesthetized with chloral hydrate (300 mg/kg, i.p.; supplemented as needed) and positioned in a stereotaxic apparatus. A heating pad and rectal thermometer system was employed to maintain body temperature at 36–38°C. A 4 mm burr hole was drilled in the skull to allow for the stereotactical guide of glass electrodes and a steel double cannula system (30-gauge syringe needles) into the right GP. Tips of both cannulas were separated 0.1 mm. The recording electrodes were placed 0.2 mm posterior to bregma, 3.0–3.3 mm lateral, and 4–6 mm ventral to brain surface according to the atlas of Paxinos and Watson [27]. The injection cannulas were placed at an angle of 70° in the latero-medial direction to the following coordinates: 1.1 mm posterior to bregma, 5.7 mm lateral, 5.6 mm ventral to the brain surface. Bethanechol and oxotremorine were used as muscarinic agonists. Atropine was employed as muscarinic antagonist. Tacrine, a selective acetylcholinesterase inhibitor, was locally applied to disclose any tonic cholinergic input to the GP. All drugs were purchased from Sigma-Aldrich, USA; drugs were dissolved in saline 0.9% and infused into the GP using a 1 *μ*L syringe. The piston of the syringe was connected to a precision micrometer that allowed an infusion rate of 50 nL/15 s. The total volume injected per individual drug infusion was 100 nL. Only neurons with five minutes of stable baseline firing were chosen for drug application. For any individual neuron, a 20% change of the baseline firing rate during the minute following drug infusion was considered significant [28]. The tips of the recording microelectrode and injection cannula were within a range of not greater than 0.2 mm [14].

### 2.2. Data Acquisition and Analysis

Standard extracellular recordings were made using glass electrodes (2–6 MΩ) filled with 2 M NaCl solution containing 2% pontamine sky blue dye. Extracellular signals were amplified 10,000 X, bandpass filtered between 0.3 and 3 kHz (DAM-80 amplifier; WPI Saratosa, Fla, USA) and stored on an audiocassette device. Single-unit activity was isolated using a window discriminator WPI-121 (WPI Saratosa, Fla, USA). Spike times were preprocessed online and further analyzed offline using the INF-386 program for spike data analysis [29]. Statistical comparisons between or among groups were determined with Student's *t*-test and one-way ANOVA (with Newman-Keuls post-hoc test) using Prism 3 (GraphPad Software). Data are expressed as the mean ± standard error or as a percentage of the control value (baseline activity).

### 2.3. Histology

At the end of each recording session, the level of the studied unit was marked by ejecting pontamine sky blue from the electrode by passing a 10 *μ*A negative constant current for 20 minutes. After experiments, rats were given a lethal overdose of pentobarbital and transcardiacally perfused with 4% formaldehyde. Following overnight incubation in formaldehyde, the brains were sliced (20 *μ*m thick slices) to verify the positions of the recording electrode and the injection cannulas. When either the recording electrode or the injection cannulas were not positioned within the GP, the experiment was discarded.

### 2.4. Striatal Lesions

Unilateral stereotaxic lesions of the corpus striatum were made in male Wistar rats (160–170 g, body weight) anaesthetized with chloral hydrate (300 mg/kg, i.p.). A 2 mm burr hole was drilled in the skull to allow for the stereotactical guide of a 30-gauge cannula into the striatum. The stereotaxic coordinates to the striatum were 1.3 mm anterior to bregma, 3 mm lateral to midline, and 4.8 mm ventral to dura [27]. The injection cannula was connected to a manual 5 *μ*L Hamilton syringe through a polyethylene plastic tube. A total of 1 *μ*L (120 nmol) of quinolinic acid was infused over 4 min. At the end of the manual infusion, the cannula was left in place for 5 more minutes to improve solution delivery. Quinolinic acid was first dissolved in 4 M NaOH and subsequently titrated with 1 N HCl to pH 7.4. A sham group of rats was prepared using the same procedure.

### 2.5. Behavioral Testing

To evaluate the lesions, rats were tested for lesion-induced turning behavior with systemic application of apomorphine (0.5 mg/kg, i.p.) 10 days after the quinolinic acid lesion. The number of ipsilateral turns was recorded and only rats showing more than 20 whole-body turns/10 minutes were considered successfully lesioned and further used for ipsilateral GP extracellular recordings. The sham group of rats was also challenged with the same dose of apomorphine. In both successfully lesioned and sham rats, extracellular recordings of ipsilateral GP neurons were made 7 days after turning behavior analysis. Single-unit extracellular recordings were made as previously described in this work. At the end of each experiment, rats were given a lethal overdose of pentobarbital and transcardiacally perfused with 4% formaldehyde. The brains were then removed and left for overnight incubation. Thereafter, the brains were sliced (20 *μ*m thick slices) to evaluate the striatal lesion as a function of the right lateral ventricle area as well as to properly determine the recording sites and drug injection cannula trajectories. When either the recording electrode or the injection cannula was not positioned within the GP, the experiment was discarded.

## 3. Results

All the spikes recorded in this study belong to type II neurons (biphasic positive/negative waveforms [28]). The spontaneous firing rate of 97 neurons recorded from normal rats ranged from 6.3 to 98.7 spikes/s. The average spiking rate was 27.3 ± 2.64 spikes/s.

### 3.1. Effect of Local Activation of MRs on the Baseline Spiking Rate

To analyze the effect of the activation of MRs on the spiking rate of GP neurons in normal rats, we locally applied different doses of bethanechol, a muscarinic agonist. All doses of bethanechol produced heterogeneous effects (Table 1). Twenty neurons were recorded in the presence of 100 pmol bethanechol, 11 (55%) increased their firing rate by 51.44 ± 3.27%, 7 (35%) diminished their firing rate by 46.05 ± 5.7% and the remaining two neurons did not respond.

**Figure 1:**
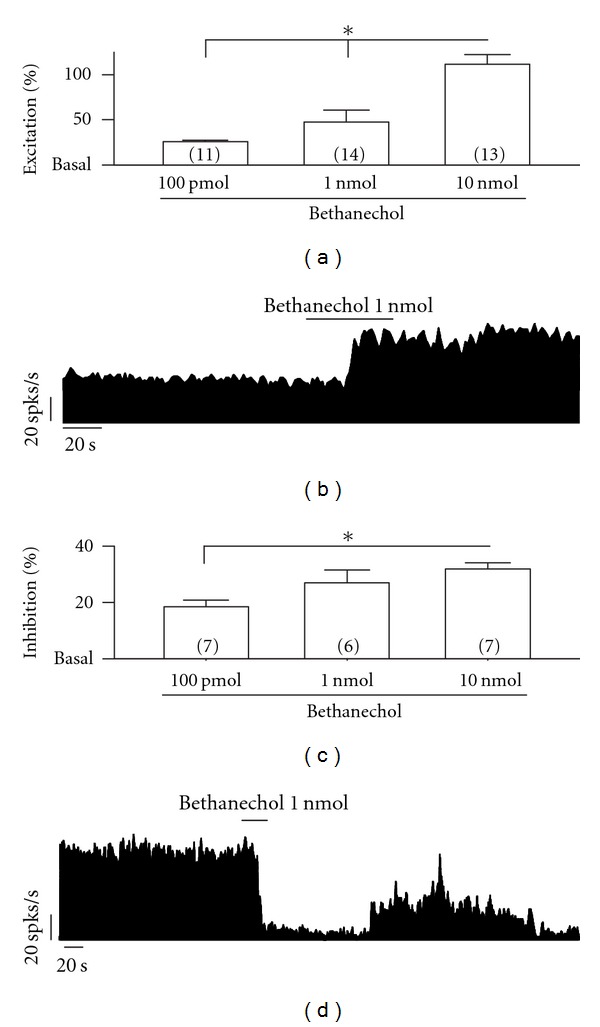
Intrapallidal activation of MRs produces heterogeneous effects on the baseline firing rate of globus pallidus neurons recorded in normal rats. (a) Bethanechol 100 pmol, 1 and 10 nmol increases the spiking rate in a dose-dependent manner (*P < 0.05 as compared to bethanechol 100 pmol and 1 nmol. One-way ANOVA, Newman-Keuls post-hoc test). (b) Frequency histogram showing the increase in baseline firing rate induced by local application of 1 nmol bethanechol. (c) In another group of globus pallidus neurons, bethanechol decreases the spiking rate in a dose-dependent manner. (*P < 0.01, as compared to 100 pmol bethanechol, one-way ANOVA, Newman-Keuls post-hoc test). (d) Frequency histogram illustrating the inhibitory effect of 1 nmol bethanechol on a pallidal neuron. spks/s, spikes per second.

Application of 1 nmol bethanechol increased the spiking rate by 94.96 ± 25.78% in 14 out of 22 neurons (64%). Six out of 22 neurons (27%) showed a 67.39 ± 11.37% inhibition in their firing rate, and the remaining two neurons (9%) did not respond (Figures 1(b) and 1(d)).

 When we applied an infusion of 10 nmol bethanechol to a group of 24 neurons, 13 (54%) increased their firing rate by 223.47 ± 20.8%, seven (29%) decreased their firing rate by 79.45 ± 5.5%, and four (17%) did not respond.

The excitatory effect evoked by bethanechol was dose-dependent. 10 nmol bethanechol caused significant differences in the firing rate when compared to 100 pmol and 1 nmol (*P* < 0.05; one-way ANOVA post-hoc test; Figure 1(a)). In addition, the inhibitory effect of bethanechol was also dose-dependent. 10 nmol bethanechol was statistically different compared to 100 pmol (*P* < 0.05; one-way ANOVA post-hoc test; Figure 1(c)).

**Figure 2:**
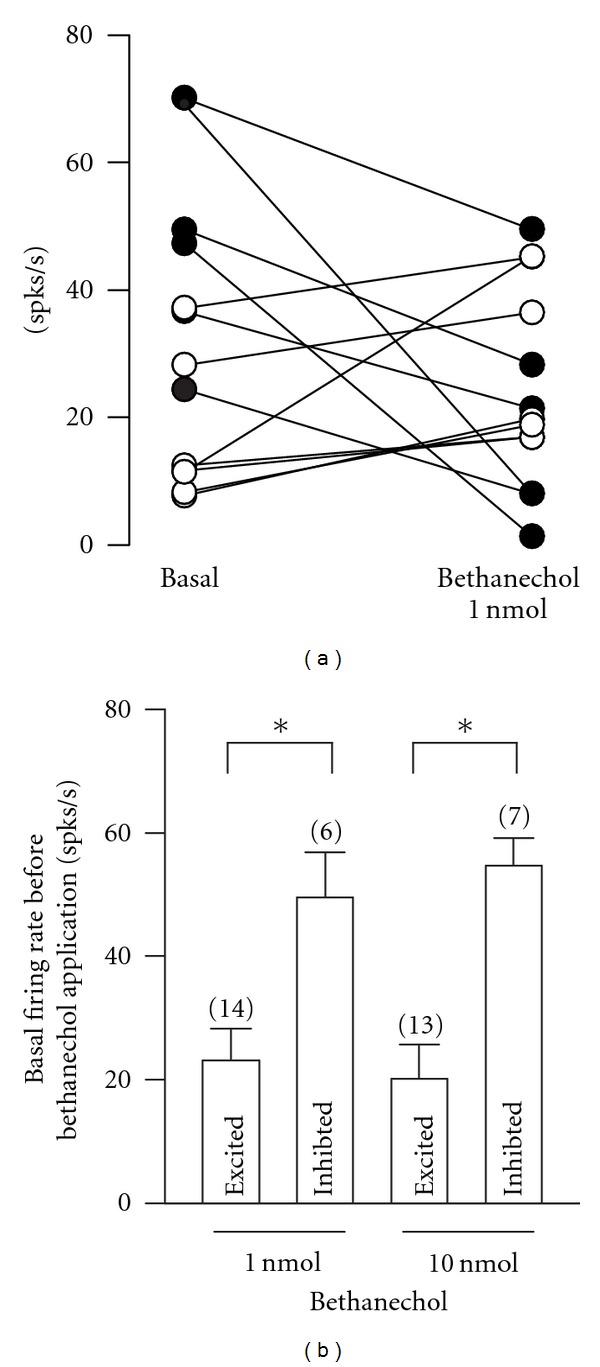
The effect of local activation of MRs depends on the firing rate before the application of different doses of bethanechol. (a) Shows that bethanechol 1 nmol produces heterogeneous effects on the spiking rate of globus pallidus neurons depending on the baseline firing rate before drug application. Only data from six neurons that suffered excitation are plotted. (b) Statistics showing that the excitation or inhibition evoked by 1 and 10 nmol bethanechol depends on the predrug baseline firing rate. *P < 0.01; *t*-test. spks/s, spikes per second.

A detailed analysis of the firing rate before and after local infusion of 1 nmol bethanechol revealed that neurons that were inhibited had a higher baseline firing rate compared to neurons that were excited or did not respond to bethanechol (Figure 2). In the six neurons that were inhibited by 1 nmol bethanechol, their baseline firing rate was 49.51 ± 7.33 spikes/s. In contrast, the baseline spiking rate in neurons that were excited by (*n* = 14) or did not respond to (*n* = 2) the same concentration of bethanechol was 23.05 ± 5.12 and 17.86 ± 1.48 spikes/s, respectively.

The baseline firing rate in neurons that were inhibited by 1 nmol bethanechol was statistically different when compared to the baseline firing rate of neurons that were excited (*P* < 0.01; *t*-test). Similarly, in the seven neurons that were inhibited by 10 nmol bethanechol, the baseline firing rate (54.72 ± 4.33 spikes/s) was higher when compared to the values of neurons that were exited (20.14 ± 5.49 spikes/s; *P* < 0.01, *t*-test). There was no statistically significant difference between the baseline firing rate of neurons that were inhibited and those that did not respond to 10 nmol bethanechol.

### 3.2. Effect of Local Blockade of MRs on the Baseline Spiking Rate

**Figure 3:**
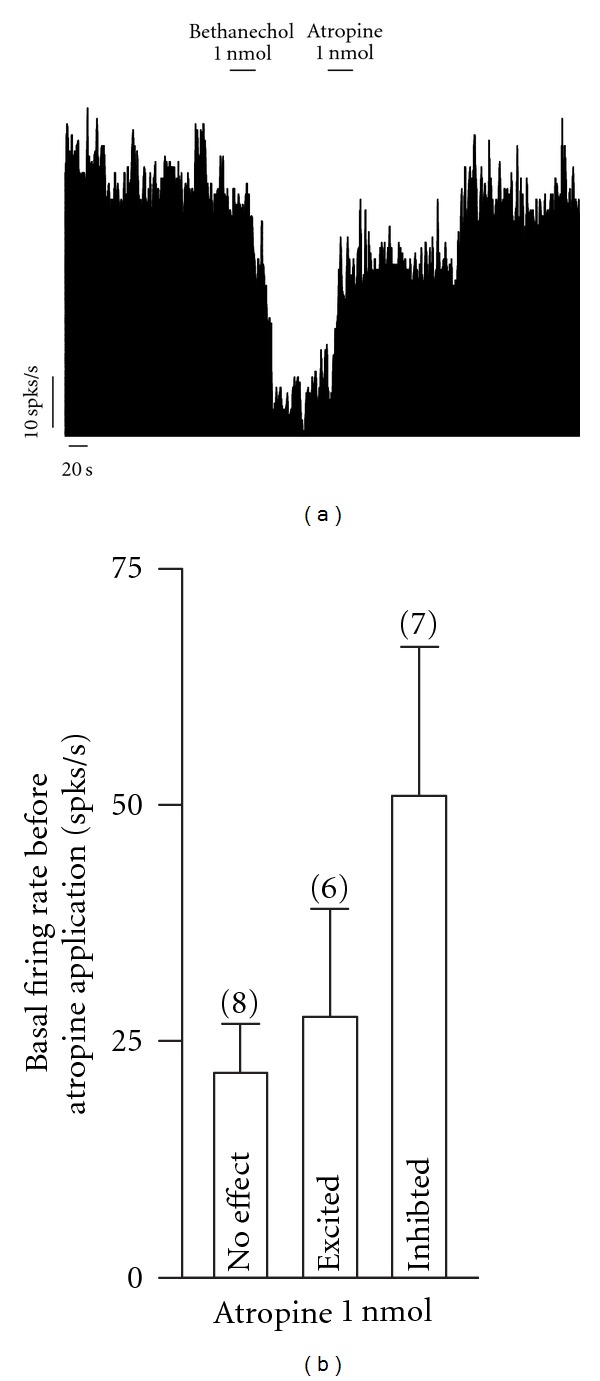
The heterogeneous effects in the spiking rate of globus pallidus neurons evoked by local blockade of MRs do not depend on the firing rate before application of atropine. (a) Frequency histogram of a pallidal neuron showing the blockade of the inhibition evoked by activation of MRs by atropine. spks/s, spikes per second. (b) Statistics illustrating that the effect of the local blockade of MRs in the spiking rate does not depend on the baseline firing rate before local infusion of 1 nmol atropine.

The effect of the local infusion of 1 nmol atropine, an unspecific muscarinic antagonist, was tested in 21 neurons. In 6 out of 21 (33%) neurons, atropine increased the spontaneous firing rate by 163.69 ± 85.5%, in this group of neurons, the spontaneous firing rate previous to the application of the antagonist was 27.6 ± 11.32 spikes/s. In eight out of 21 neurons (38%) atropine did not produce significant changes (−4.25 ± 5.09%). In neurons that did not respond to atropine, the baseline firing rate was 21.68 ± 5.18 spikes/s. In seven out of 21 neurons (33%), local infusion of atropine diminished their baseline firing rate by 48.19 ± 10.72%, the baseline firing rate of this group of neurons before local application of atropine was 50.97 ± 15.71 spikes/s. There was not statistically significant difference between the baseline firing rate of those neurons that were inhibited and those that did not respond or were excited by atropine (one-way ANOVA post-hoc test; Figure 3(b)). In some neurons, the firing rate changes evoked by bethanechol were blocked by atropine (Figure 3(a)).

### 3.3. Effect of Local Blockade of Acetylcholinesterase on the Baseline Spiking Rate

**Figure 4:**
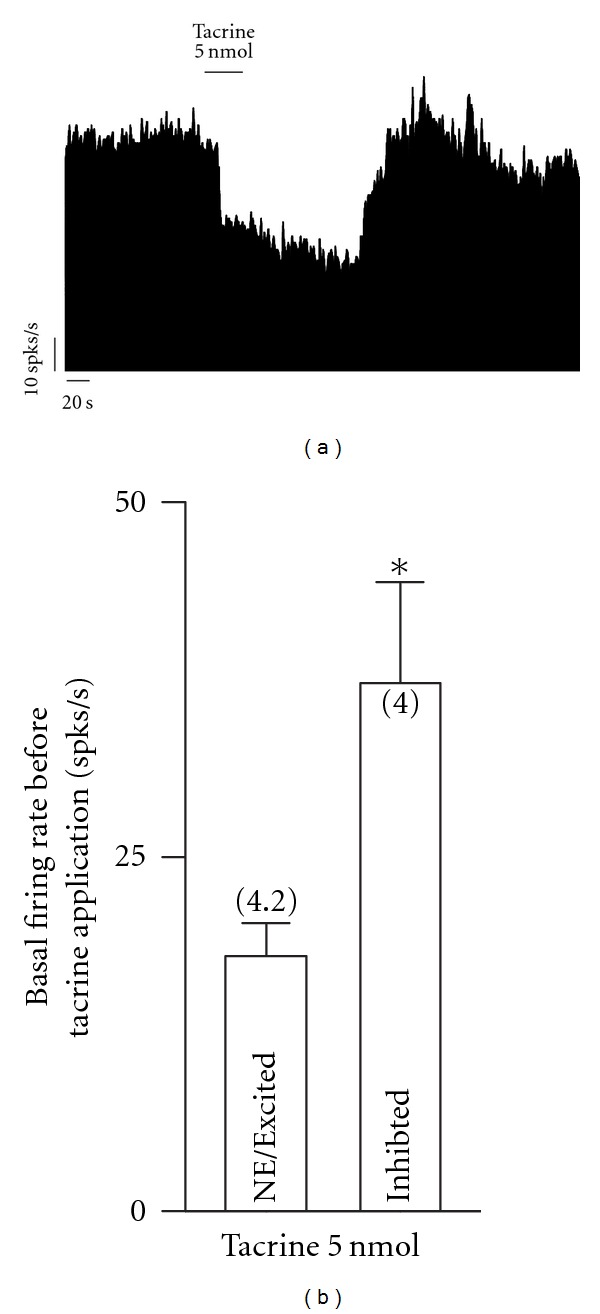
The increase in the tonic cholinergic input modifies the firing rate in globus pallidus neurons depending on the baseline firing rate before application of tacrine. (a) Frequency histogram showing the firing rate inhibition induced by local application of 5 nmol tacrine in a globus pallidus neuron firing at ~75 spikes/s. (b) Statistics illustrating that the effect of the local blockade of acetylcholinesterase in the spiking rate depends on the baseline firing rate before local infusion of 5 nmol tacrine (*P < 0.01; *t*-test). spks/s, spikes per second; NE/Excited, no effect or excitation.

To determine if there is a tonic release of acetylcholine in the GP, we locally applied tacrine, a selective inhibitor of acetylcholinesterase. Tacrine (5 nmol) evoked heterogeneous responses in the firing rate of 10 GP neurons: two (20%) were excited by 58.9 ± 24.42%, four (40%) were inhibited by 67.38 ± 17.27%, and the remaining four neurons (40%) did not respond. The baseline spiking rate in neurons that were inhibited by tacrine was 37.24 ± 7.17 spikes/s, while the baseline firing rate in neurons that were excited or did not respond was 19.85 ± 0.21 and 20.32 ± 0.57 spikes/s, respectively. There were statistically significant differences between the baseline firing rate of neurons that were inhibited and those that were exited or did not respond to tacrine (*P* < 0.01; *t*-test; Figure 4).

### 3.4. Effect of Local Activation of MRs by Bethanechol in Rats with Ipsilaterally Denervated Striatum

To analyze the contribution of the striato-pallidal pathway on the local responses evoked by MRs activation, we proceeded to destroy the ipsilateral striatum with quinolic acid. To test if the ipsilateral striatum was properly destroyed, we analyzed the rotational behavior by applying apomorphine 0.5 mg/kg (i.p.) one week after the infusion of the neurotoxic.

Rats that presented 20 whole-body turns/10 minutes were chosen to analyze the spiking activity of GP neurons. A sham group of four rats was prepared applying the same volume of saline.

**Figure 5:**
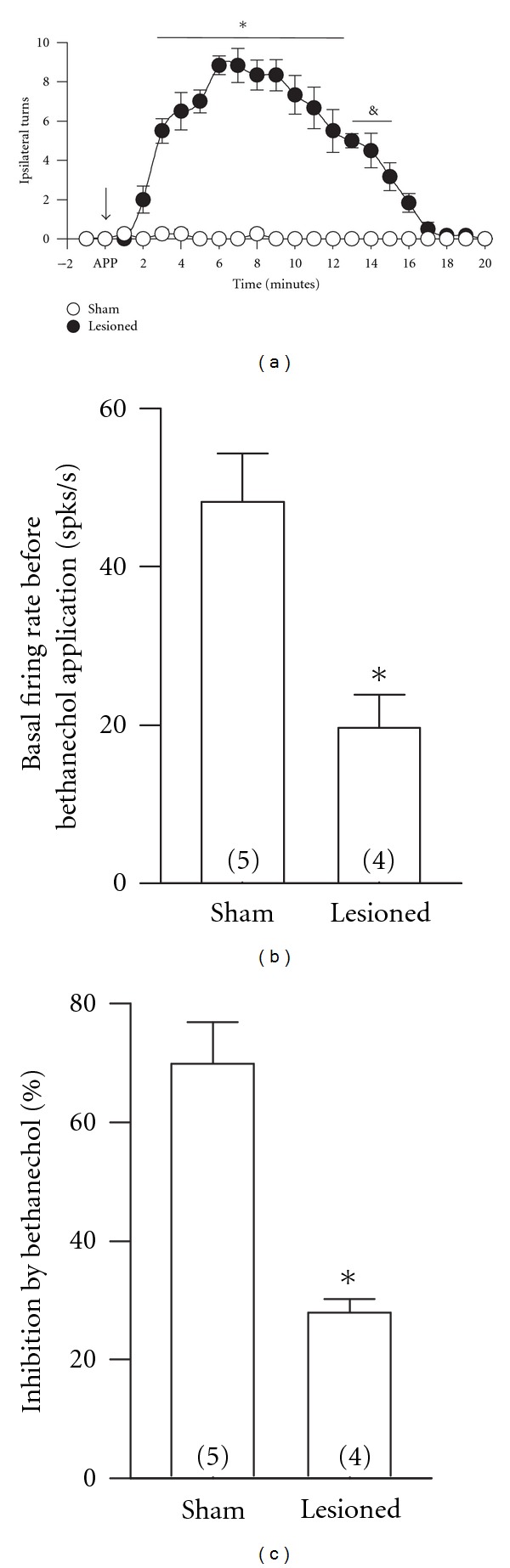
The activation of MRs in the globus pallidus has differential characteristics between sham and ipsilateral striatum-lesioned rats. (a) Intraperitoneal application of apomorphine (0.5 mg/kg) induces ipsilateral turning behavior in rats with striatal lesion. (*P < 0.01; & *P* < 0.05, *t*-test). (b) In sham rats, the predrug (1 nmol bethanechol) baseline firing rate was higher in neurons that were inhibited by bethanecol than the baseline firing rate recorded in neurons from lesioned rats that were also inhibited by the same concentration of bethanechol (*P < 0.029, *t*-test). (c) The percent of inhibition evoked by 1 nmol bethanechol is higher in neurons recorded in sham rats than in those recorded in lesioned rats (*P < 0.04, *t*-test). APP, application time (down arrow); spks/s, spikes per second.

We recorded GP neurons in the ipsilaterally denervated striatum five days after the rotational behavior test (Figure 5(a)). The mean baseline firing rate of 28 neurons recorded in lesioned rats was 22.33 ± 2.1 spikes/s (range: 5.27–68.6 spikes/s). The local infusion of 1 nmol bethanechol produced heterogeneous effects in GP neurons of lesioned rats (Table 2). In 19 out of 28 (68%) neurons recorded from seven lesioned rats, bethanechol increased the firing rate by 102 ± 27%, and only in four (14%) neurons bethanechol diminished the firing rate by 27.8 ± 3.36%. In the remaining five neurons (18%) bethanechol had no effect. Further, 19 neurons were recorded in the sham group. Eleven out of 19 (58%) were excited 87.2 ± 17.3% by the same dose of bethanechol used in lesioned rats. Five out of 19 neurons (26%) diminished the basal firing rate by 55.3 ± 9.1%. The remaining tree neurons (16%) did not respond to bethanechol.

In neurons recorded from lesioned rats, the baseline firing rate of those cells that were inhibited by 1 nmol bethanecol was 22.54 ± 9.27 spikes/s whereas in those that were excited by and in those that did not respond to bethanechol was 20.8 ± 3.9 and 22.15 ± 3.22 spikes/s, respectively. There were no statistically significant differences in the baseline firing rate of neurons recorded from lesioned rats independently of the effect of bethanechol. In the sham group of rats, the baseline spiking rate of the neurons that were inhibited was 48.2 ± 6 spikes/s, whereas in those neurons that were excited or did not respond, the baseline firing rate was 24 ± 5.3 and 21.5 ± 3.7 spikes/s.

### 3.5. Differences in the Response to the Activation of MRs in Sham and Lesioned Striatum

In sham rats, the baseline firing rate was higher in neurons that were inhibited by 1 nmol bethanechol than in neurons of lesioned rats that were also inhibited (*P* < 0.01, *t*-test; Figure 5(b)). The percent of inhibition evoked by 1 nmol bethanechol in neurons recorded in sham rats was higher than the percent of inhibition in lesioned rats (*P* < 0.01, *t*-test; Figure 5(c)). There was no significant difference in the excitation induced by 1 nmol bethanechol in both groups of rats. The percentage of neurons that were inhibited by the same dose of bethanechol was lower when compared to sham rats.

### 3.6. Effect of Oxotremorine on the Spiking Rate of GP Neurons of Sham and Ipsilaterally Denervated Striatum Rats

Finally, we analyzed the effect of another muscarinic agonist on the spontaneous firing rate of GP neurons in both sham and lesioned rats. We employed oxotremorine because the affinities of bethanechol and oxotremorine for M1 and M4 receptors are similar [30]. The baseline firing in sham rats and lesioned rats were 23.6 ± 8.3 and 25.9 ± 1.8 spikes/s, respectively. Oxotremorine (1 nmol) produced heterogeneous effects in sham rats: in seven out of 13 neurons (54%) induced a 64.49 ± 27% increase and two neurons (16%) did not respond. In neurons that increased their firing and in neurons that did not respond, the baseline firing rate was 27 ± 3.1 and 22.45 ± 2.3 spikes/s, respectively. In four neurons (30%) the firing rate diminished by 33 ± 3.4%. The spontaneous baseline firing rate in neurons that diminished their firing was 42.73 ± 15.4 spikes/s. Whereas in lesioned rats, the same dose of oxotremorine produced a 92 ± 12.7% increase in the baseline firing rate of seven out of 11 neurons tested (64%). In two out of 11 neurons (18%) oxotremorine diminished the firing rate by 30.5 ± 2.2%. In the remaining two neurons (18%) oxotremorine had no effect. There was a significant difference in the inhibition induced by 1 nmol oxotremorine in both groups of rats (*P* < 0.01, *t*-test, Table 3).

## 4. Discussion

Previous works have demonstrated that the GP is innervated by cholinergic pathways coming from the PPN [17, 18, 20], a few pallidal neurons that express choline acetyltransferase together with glutamic acid decarboxylase that weights 67 kDa (GAD67) [31], and the presence of MRs in the GP [21, 22] but there are few studies in which the functional role of the MRs in GP has been analyzed. Moreover, previous works have found mRNA for M1 and M4 receptors in medium spiny striatal neurons and the expression of M1 and M4 muscarinic receptor proteins in the striatum but have not detected mRNA for MRs in GP neurons [23–25, 32, 33]. This is the first study where the contribution of MRs to the spiking activity of GP neurons recorded *in vivo* both in normal and ipsilateral striatum-lesioned rats is analyzed. Our results indicate that local activation or blockade of MRs produces heterogeneous effects in the spontaneous spiking activity of GP neurons of both groups of rats. In the one hand, in normal rats, the response of pallidal neurons evoked by activation of MRs depends on the baseline firing rate before local application of drugs; neurons with high baseline spiking rates diminish their activity, whereas those with lower spiking rates increase their activity or do not change. In the other hand, in pallidal neurons recorded from ipsilateral striatum-lesioned rats, we did not find any relationship between the basal firing rate before and after activation of MRs.

We demonstrate the presence of a tonic release of acetylcholine in the GP linked to the activity of MRs since if we block MRs with atropine or inhibit acetylcholinesterase, the spontaneous spiking rate significantly changes in most of the recording neurons.

Kayadjanian et al. [26] reported that in rat brain slices the application of a muscarinic agonist increases GABA release in the GP through phosphoinositides and that the effect is blocked by a muscarinic antagonist. These results were attributed to the activation of presynaptic MRs in the striato-pallidal pathway; however, there is not a direct demonstration of the presence of these receptors in pallidal terminals of medium spiny neurons. In this context, the activation of striatal M1 receptors diminishes a variety of calcium conductances including N and P/Q currents in the dendrites and soma of medium spiny neurons [34]. N and P/Q currents are associated with neurotransmitter release through activation of Gq/11-PLC [35, 36]. The functional role of striatal M4 receptors is yet to be determined [37].

In our work, there were some differences in the response of MRs in normal and ipsilateral striatum-lesioned rats. Besides the relationship between the effect and the basal spiking activity previous to the activation of MRs, we found that (1) the percent of inhibition evoked by the activation of MRs in normal rats was higher than in lesioned rats and (2) the number of neurons that were inhibited in lesioned rats was lower than in normal rats.

Therefore, our data show the contribution of the striato-pallidal pathway to the responses evoked by local activation of MRs in the GP. Supporting this hypothesis, the expression of MRs on GABAergic terminals in many regions of the CNS is common, but the regulation of GABA release by these receptors is more complex; for example, Baba et al. [38] have pointed out that activation of MRs increases GABA release in the substantia gelatinosa of the rat spinal horn; Grillner et al. [39] reported similar results in mesencephalic dopaminergic nuclei, while in the rat subfornical organ, bethanechol blocks GABA release [40]; in a similar manner, it has been found that activation of MRs increases or diminishes GABA release in the chick lateral spiriform nucleus [41].

In our work, the heterogeneous responses evoked by activating local MRs in normal and lesioned rats can be explained according to the differential segregation of M1 and M4 receptors in different pools of striato-pallidal terminals. Differential distribution of MRs in striatal medium spiny neurons is considerable; 100% of medium spiny enkephalinergic neurons express M1 receptors while 30–50% of this kind of neurons expresses M4 receptors [23, 25]. M1 receptors diminish GABA release in striatal neurons [34] but the effect of M4 receptors on medium spiny neurons is unknown, although presynaptic activation of M4 receptors in the spinal dorsal horn and in the hippocampus increases GABA release [42, 43].

Nevertheless, the differential expression of presynaptic M1 and M4 receptors does not fully explain why in normal rats exists a direct relationship between the baseline spiking rate level and the effect caused by the drugs we tested. There is not an easy explanation of this phenomenon. An idea would be that in pallidal neurons firing at low frequency, the striato-pallidal terminals could express M4 receptors while pallidal neurons with higher spontaneous frequency could express M1 receptors. Segregation of M1 and M4 receptors in striato-pallidal terminals could depend on the basal activity of postsynaptic events. In agreement with this idea, using triple-whole cell patch-clamp recording from GABAergic interneurons in the rat insular cortex, it has been shown that carbachol diminishes or augments the amplitude of inhibitory postsynaptic currents (IPSCs) activating M1 or M4 receptors in different terminals of the same cell. Expression of M1 or M4 depends on the type of postsynaptic neuron [44]; furthermore, three different types of GP neurons have been characterized based on their membrane properties [45, 46]. However, we concede that single-unit extracellular recordings cannot readily differentiate these types of neurons. In this context, one could not discard the expression of MRs in another afferents, like glutamatergic fibers coming from the subthalamic nucleus that play a major role in globus pallidus activity [47]. Further, activation of M3 receptors modifies the activity of subthalamic neurons [48] but M3 receptors are expressed on afferents fibers and have not been detected in subthalamic neurons [49].

Our results show a tonic cholinergic activity in the GP. It has been shown in preparations similar to ours that neurons of the PPN fire spontaneously [50, 51]. This spontaneous activity of PPN neurons would explain the presence of the tonic cholinergic release into GP reported here. This cholinergic input could be mediated by MRs as our data on the local blockade of MRs points out. However, we must consider that there are few cholinergic neurons in the medial border of the GP near to the basalis nucleus of Meynert and that their contribution to the whole GP activity is unknown [31]. Amacrine cells in the retina corelease GABA and acetylcholine but the stimuli and mechanisms involved are quite complex [52].

Finally, with the destruction of the striato-pallidal fibers, one would expect an increase in the spiking rate of GP neurons of lesioned rats [53, 54] but there are no differences between the baseline firing rate recorded in sham rats and that recorded in ipsilateral striatum-lesioned rats; one possible explanation to this is that chloral hydrate as anesthetic modifies the GABAergic transmission as compared to locally anesthetized rats [55]. Despite studies showing the presence of mRNA for M1 and M4 receptors in striato-pallidal neurons, the location of MRs in pallidal neurons should not be disregarded; some of the effects reported here could be due to postsynaptic MRs that until now have not been detected.

## 5. Conclusion

Our data indicate that MRs homogenize the activity of pallidal neurons; neurons with high spiking rates diminish their firing frequency and those with low activity increase it. A similar observation was made in the striatum: activation of MRs in the striatum synchronizes the activity of ensembles of different groups of medium spiny neurons [56]. The effect of MRs on GP neurons depends on their basal activity and on the integrity of the striato-pallidal pathway. Modulation of MRs in the GP could be a pharmacological target to improve some motor system alterations.

## Figures and Tables

**Table 1:** Response of GP neurons to different concentrations of bethanechol.

	Increase	Decrease	No response	Total
100 pmol	11 (55%)	7 (35%)	2 (10%)	20 (100%)
1 nmol	14 (64%)	6 (27%)	2 (9%)	22 (100%)
10 nmol	13 (54%)	7 (29%)	4 (17%)	24 (100%)

Values represent *n* (%).

**Table 2:** Response of GP neurons to 1 nmol bethanechol in normal and ipsilateral striatum-lesioned rats.

	Increase	Decrease	No response	Total
Normal	11 (58%)	5 (26%)	3 (16%)	19 (100%)
Lesioned	19 (68%)	4 (14%)	5 (18%)	28 (100%)

Values represent *n* (%).

**Table 3:** Response of GP neurons to 1 nmol oxotremorine in normal and ipsilateral striatum-lesioned rats.

	Increase	Decrease	No response	Total
Normal	7 (54%)	4 (30%)	2 (16%)	13 (100%)
Lesioned	7 (64%)	2 (18%)	2 (18%)	11 (100%)

Values represent *n* (%).
